# High Seroprevalence of *Toxoplasma gondii* and *Neospora caninum* Infections Among Goats in Mexico Is Associated with Climatic, Environmental, and Risk Factors

**DOI:** 10.3390/pathogens14111170

**Published:** 2025-11-16

**Authors:** Abel Villa-Mancera, Eunice Vargas-Tizatl, José Manuel Robles-Robles, Fernando Utrera-Quintana, Jaime Olivares-Pérez, Agustín Olmedo-Juárez, Alejandro Córdova-Izquierdo, Roberto González-Garduño, José Luis Ponce-Covarrubias, Nallely Rivero-Perez, Felipe Patricio-Martínez, Huitziméngari Campos-García

**Affiliations:** 1 Facultad de Medicina Veterinaria y Zootecnia, Benemérita Universidad Autónoma de Puebla, Tecamachalco 75482, Puebla, Mexico; vt223461997@alm.buap.mx (E.V.-T.); manuel.roblesr@correo.buap.mx (J.M.R.-R.); fernando.utrera@correo.buap.mx (F.U.-Q.); felipe.patriciomtz@correo.buap.mx (F.P.-M.); huitzi.campos@correo.buap.mx (H.C.-G.); 2 Programa de Maestría en Producción Animal Sostenible, Benemérita Universidad Autónoma de Puebla, Tecamachalco 75482, Puebla, Mexico; 3 Unidad Académica de Medicina Veterinaria y Zootecnia, Universidad Autónoma de Guerrero, Ciudad Altamirano 40662, Guerrero, Mexico; jaimeolivares@uagro.mx; 4 Centro Nacional de Investigación Disciplinaria en Salud Animal e Inocuidad (CENID SAI-INIFAP), Jiutepec 62550, Morelos, Mexico; olmedo.agustin@inifap.gob.mx; 5 Departamento de Producción Agrícola y Animal, Universidad Autónoma Metropolitana, Unidad Xochimilco, CDMX 04960, Mexico; acordova@correo.xoc.uam.mx; 6 Unidad Regional Universitaria Sursureste, Universidad Autónoma Chapingo, Teapa 86800, Tabasco, Mexico; rgonzalezg@chapingo.mx; 7 Escuela Superior de Medicina Veterinaria y Zootecnia No. 3, Universidad Autónoma de Guerrero, Técpan de Galeana 04960, Guerrero, Mexico; jlponce@uagro.mx; 8 Área Académica de Medicina Veterinaria y Zootecnia, Instituto de Ciencias Agropecuarias, Universidad Autónoma del Estado de Hidalgo, Pachuca de Soto 40900, Hidalgo, Mexico; nallely_rivero@uaech.edu.mx

**Keywords:** *Toxoplasma gondii*, *Neospora caninum*, seroprevalence, ELISA, risk factors, goats, geographical information system, epidemiology, Mexico

## Abstract

*Toxoplasma gondii* and *Neospora caninum* are intracellular protozoan parasites that cause reproductive failure and production losses in ruminants. Considering the limited information on the epidemiology of these infections in goats in different climate regions, this study aimed to estimate the seroprevalence and potential risk factors associated with parasitic infections in Mexico. Blood samples were collected from 627 goats in dry and temperate climates in two different states. The levels of *T. gondii* and *N. caninum* IgG antibodies were determined using commercially available ELISA kits. The prevalence of *T. gondii* in the dry and temperate climate, dry climate alone, and temperate climate alone were 52.0%, 57.1%, and 48%, respectively. The prevalence of *N. caninum* in the dry and temperate climate, dry climate alone, and temperate climate alone were 15.5%, 19.0%, and 12.7%, respectively. Using animal characteristics and farm management information obtained from a questionnaire and remotely sensed climate data, bivariate logistic regression analysis was performed to identify risk factors associated with parasite infections. Significant differences in the seroprevalence of *T. gondii* in goats were observed between sexes in the temperate climate. The history of abortion was the most significant risk factor for *T. gondii* in the dry climate. Factors such as goat age and history of abortion were significantly associated with high seropositivity of *N. caninum* in the dry climate. Sex and the presence of cats were identified as significant factors for *T. gondii* in regions with a dry and temperate climate. Abortion and climate regions were common risk factors for these infections in the dry and temperate climate regions. The results indicate that regionally adapted monitoring and control programmes may be developed to reduce the prevalence of these two parasites and reduce production losses in the livestock industry.

## 1. Introduction

*Toxoplasma gondii* and *Neospora caninum* infections are important causes of abortion worldwide [1,2]. Economic losses due to toxoplasmosis and neosporosis are estimated to be US$1.4–4.7 million in Uruguay and US$1.298 billion annually [3,4], respectively. Domestic and wild felids are the definitive hosts of *T. gondii*, while dogs, wolves, coyotes, and dingoes are the definitive hosts of *N. caninum.* Both parasites affect a wide range of intermediate hosts, particularly cattle, sheep, and goats [1]. Both parasites infect the host horizontally through environmental contamination caused by the definitive hosts shedding oocysts in their faeces or transplacental transmission from the dam to the foetus [5,6].

The epidemiological patterns of foodborne parasites transmitted by both domesticated and wild animals are susceptible to changes in animal biomass, migration, and human introduction. Consequently, globalisation and climate change are predicted to significantly alter these pathogen dynamics and ecosystems by modifying biotic and abiotic factors [7]. Felids excrete millions of oocysts into the environment, with the sporulation, survival, and infectivity of these oocysts contingent on environmental and climatic conditions [8,9]. Other studies indicate that environmental risk factors and geoclimatic factors are significantly associated with the distribution of parasite infection [10]. The risk factor categories, which include individual animal characteristics, farm management, climate, and the environment, provide parameters that are significantly related to the occurrence of *N. caninum* [11].

Geographical information systems (GIS) and remote sensing technologies are useful and convenient tools in modern disease surveillance, globally providing essential, comprehensive, and updated information on the presence of economically important infections and risk factors, improving the existing strategies for preventing and controlling disease [12].

The worldwide estimated seroprevalence of *T. gondii* and *N. caninum* in goats is 27.49% and 5.99% [6,13], respectively. However, only a limited number of studies in goats have reported the prevalence and risk factors of both pathogens in the different regions of Mexico, which have dramatically different climatic conditions. The objectives of the present study were to (i) investigate the prevalence of *T. gondii* and *N. caninum* in dry and/or temperate climates in two different Mexican states and (ii) perform bivariate logistic regression analysis using climatic, environmental, and management data to identify potential risk factors for parasite infections.

## 2. Materials and Methods

### 2.1. Study Area and Sample Collection

This study protocol was conducted according to the guidelines of the Animal Care and Ethics Committee of the Meritorious Autonomous University of Puebla (458861), and included the handling and collection of blood samples in accordance with the national legislation of animal health research.

**Figure 1:**
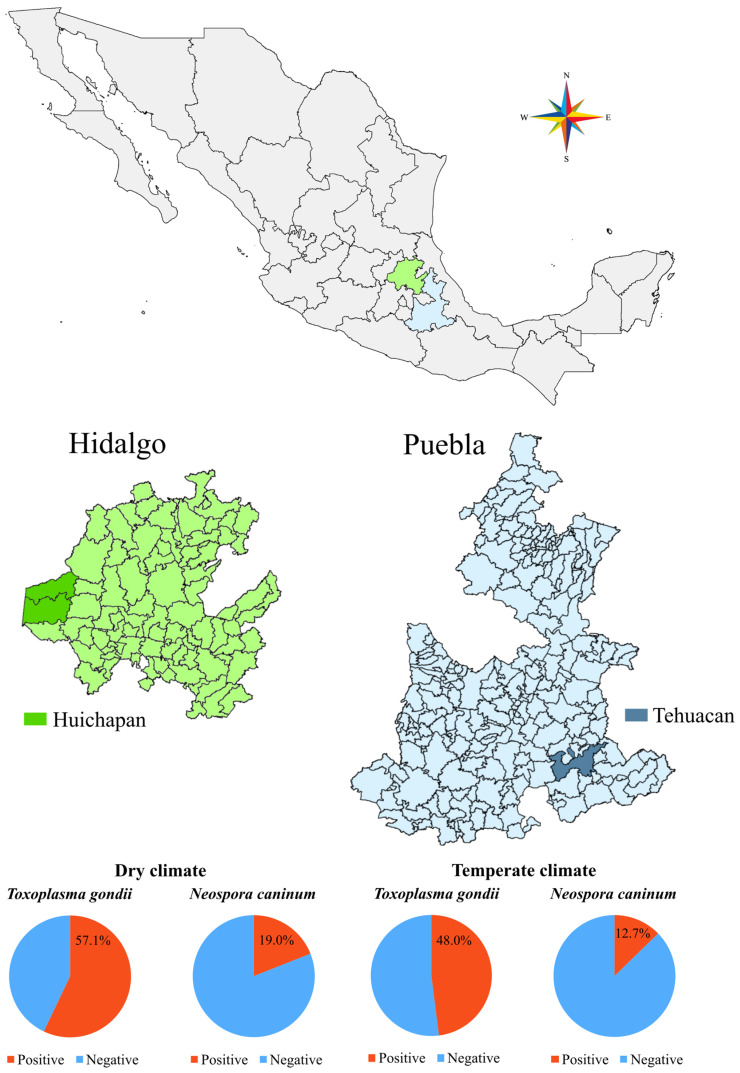
Geographical location of the states of Hidalgo (Huichapan district) and Puebla (Tehuacan district) and seroprevalence of *Toxoplasma gondii* and *Neospora caninum* in two climate regions in Mexico.

The study was conducted in two different states of Mexico (Figure 1): Puebla (eastern-central Mexico, 33,919 km^2^) and Hidalgo (eastern-central Mexico, 20,987 km^2^). A cross-sectional study was performed in the district of Tehuacan (Puebla state), which includes the municipality of Tehuacan. The Huichapan district of Hidalgo state includes the municipalities of Tecozautla and Huichapan. The climate of Mexico is characterised by significant diversity, with substantial variations in temperature and precipitation throughout different regions [14]. The states are categorised into various climate groups, predominantly tropical wet (Puebla), tropical wet-and-dry (Puebla), temperate with dry winters (Puebla and Hidalgo), and semi-arid (Puebla and Hidalgo). The state’s climate includes a summer rainy season from June to September/October, with an average annual temperature ranging from 15 °C to 18 °C, and annual precipitation varying between 400 and 800 mm.

A total of 627 serum samples from different goat breeds (mainly Creole, Boer, Nubian, Saanen, and their crosses) were obtained using convenience sampling based on the willingness of the owners and managers to take part in research. The blood samples were obtained by a trained veterinarian and one member of staff, transported in an ice box to the Laboratory of Molecular Biology and Veterinary Biotechnology, Meritorious Autonomous University of Puebla. Sera were centrifuged at 2000× *g* for 10 min at 4 °C, and the sera were then transferred to cryotubes and stored at −80 °C for enzyme-linked immunosorbent assay (ELISA) analyses. From September 2024 to December 2024, an epidemiology questionnaire was used to identify the risk factors that contribute to the exposure of goats to *T. gondii* and *N. caninum* infections. The data obtained from the questionnaire, including animal identification, age (years), sex (male/female), history of abortion (no/yes), presence of resident or stray cats and dogs on the property, and rearing system (intensive/semi-intensive), were collected on an Excel spreadsheet. Goats were classified into age groups of ≤1 year, 1 to 2 years, and ≥2 years. The rearing system of surveyed farms was classified as intensive (permanent confinement and feed supplementation) and semi-intensive (daily grazing during the day and confinement during the night).

### 2.2. Remotely Sensed Climatic Data

The latitude and longitude of each farm were identified using a Garmin eTrex Vista global positioning system (GPS), and their geographical positions were georeferenced and overlaid in the GIS environment (ArcGIS 10.1 ESRI; Redlands, CA, USA) using Köppen climate classification maps modified by [14]. For meteorological data, remotely sensed climate data products with global coverage were extracted as monthly means from historical series dating from 2019 to 2024. Monthly rainfall data were acquired from the Tropical Rainfall Measuring Mission (http://disc2.gesdisc.eosdis.nasa.gov (accessed on 6 February 2025) 3B43 satellite product, featuring a spatial resolution of 0.25° × 0.25°. Land surface temperature (LST) is a crucial metric that indicates land–atmosphere interactions, and the normalised difference vegetation index (NDVI) at a spatial resolution of 0.05° was obtained from the Moderate Resolution Imaging Spectroradiometer (MODIS) Terra products, MOD11C3 v.061 and MOD13A2 v.061. LST data served as a proxy for day and night temperature, while NDVI was used as a proxy for soil moisture [15] and vegetation activity at the land surface. A 1 km resolution Digital Elevation Model Shuttle Radar Topography Mission (SRTM) dataset was used to obtain the elevation of the farms.

### 2.3. Serological Examination of T. gondii and N. caninum

In order to detect antibodies to *T. gondii*, all serum samples (*n* = 627) were analysed with the ID Screen Toxoplasmosis Indirect ELISA Multi-species kit (ID Screen, ID.vet Innovate Diagnostics, Montpellier, France), according to the manufacturer’s instructions. The indirect ELISA based on native P30 (SAG1) antigen utilised an anti-multi-species conjugated secondary antibody. The manufacturer’s data indicate that the P30 *T. gondii* antigen has a sensitivity of 100% and a specificity of 96%. The analysis of the goat serum samples, in addition to the positive and negative controls supplied in the kit, was performed in duplicate. Absorbance values were measured at 450 nm using an ELISA reader (BioTek ELx800, BioTek Instruments, Inc., Winooski, VT, USA), and the resulting values were used to calculate the percentage of sample-to-positive ratio (S/P%). Samples exhibiting S/P% values of at least 50% were considered positive, while those with values ranging from 40% to 50% were classified as doubtful and those with values below 40% were regarded as negative.

Serum samples were analysed to detect anti-*N. caninum* IgG antibodies using a commercial indirect ELISA kit (ID Screen, ID.vet Innovate Diagnostics) with a sensitivity of 100% and a specificity of 100%. Multi-species conjugates were used as secondary antibodies. The optical density at 450 nm was determined using an ELISA reader (BioTek ELx800). The results are expressed as S/P%, and according to the manufacturer’s guidelines, goats with S/P% ≥ 50% were considered positive.

### 2.4. Statistical Analysis

Data were analysed using IBM SPSS v.25 software for Windows (SPSS Inc., Chicago, IL, USA). The true prevalence was estimated using the Rogan-Gladen estimator [16] method using the specificity and sensitivity indicated by the kit for each microorganism, as claimed by the manufacturer. Bivariate logistic regression analysis was performed with the *T. gondii* and *N. caninum* infection status (seropositive/seronegative) as dependent variables and the potential risk factors from the questionnaire, as well as climatic/environmental data, as independent variables. Age, sex, history of abortion, presence of cat/dogs, and rearing system variables collected with the questionnaire were treated as categorical variables and climatic/environmental factors as numerical variables. Odds ratios (OR) are reported with 95% confidence intervals (CI).

## 3. Results

### 3.1. Seroprevalence of T. gondii in Goats

The overall seroprevalence was 52.0% (326 out of 627 samples) for IgG antibodies to *T. gondii* by ELISA; 6.1% (38 out of 627 samples) of the animals were seropositive for both pathogens. The true prevalence of *T. gondii* was 50.0%. The prevalence of goats infected with *T. gondii* in the temperate climate, as indicated by ELISA, was 48.2% (170 out of 354 samples). A summary of the serological prevalence in goats is presented in Table 1. This study shows that the true prevalence was 45.9% for IgG antibodies against *T. gondii* in the temperate climate. The *T. gondii* prevalence in the dry climate (Hidalgo) was 57.1% (156 of 273 samples). Table 2 presents a summary of the serological detection using diagnostic tests in goats. The true prevalence of animals infected with *T. gondii* based on indirect ELISA in the dry climate was 55.4%.

### 3.2. Risk Factors of T. gondii in a Temperate Climate

The seroprevalence of *T. gondii* in the different age groups ranged from 22.7% (1–2 years old) to 72.6% (≥2 years old; Table 1). The seroprevalence in goats was higher in males (61.0%) than in females (42.6%), in cases of abortion (76.8%) compared to no history of abortion (22.0%), in the presence of cats (55.5%) compared to the absence of cats (37.1%), and in semi-intensive systems (48.6%) compared to intensive systems (43.6%). In bivariate logistic regression analysis, male sex was associated with *T. gondii* seropositivity in goats (*p* = 0.002; OR 2.106; 95% CI: 1.322–3.355), whereas no statistically significant associations were found between *T. gondii* seroprevalence and age, abortion, presence of cats, rearing system, or climate and environment variables.

### 3.3. Risk Factors of T. gondii in a Dry Climate

The prevalence of *T. gondii* in goats ranged from 14.3% to 81.3% by age group in the dry climate of Hidalgo state. *T. gondii* was detected in 59.8% of males, and a lower prevalence was observed in female goats (55.1%). Detailed results are listed in Table 2. The highest prevalence for *T. gondii* was detected in goats with a history of abortion (85.9%), compared with 18.8% in goats with no history of abortion. The highest prevalence of *T. gondii* was detected in goats in contact with cats (61.7%) and those raised in a semi-intensive system (67.9%), compared with 48.4% in animals with no contact with cats and 55.9% in animals raised in an intensive system.

A history of abortion was the most significant risk factor of *T. gondii* infection in goats: animals with a history of abortion had 26.302 times higher risk of being infected than animals with no history of abortion (OR 26.302; 95% CI: 13.775–50.220). The association was lower when considering variables of age, sex, presence of cats, rearing system, climate, and the environment.

### 3.4. Risk Factors of T. gondii in Both Dry and Temperate Climates

The prevalence of *T. gondii* in temperate climates was highest among ≥2-year-olds (76.2%), followed by 1–2-year-olds (41.0%) and ≤1-year-olds (18.9%). As shown in Table 3, the seroprevalence of *T. gondii* was higher in males (58.1%) than in females (48.6%), in goats with a history of abortion (81.2%) compared to those with no history of abortion (20.8%), in animals in the presence of cats (59.25%) compared to the absence of cats (44.2%), and in intensive rearing systems (54.2%) compared to semi-intensive systems (50.1%). The highest prevalence among the two climate regions was found in the dry climate (57.1%, 156 out of 273), followed by the temperate climate (48.0%, 170 out of 354; Table 3).

From the bivariate logistic regression analysis, the variables that were significantly associated with *T. gondii* seropositivity included female sex (OR 1.465; 95% CI: 1.053–2.038), history of abortion (OR 16.425; 95% CI: 11.086–24.335), presence of cats (OR 1.970; 95% CI: 1.419–2.734), and the temperate climate in Puebla state (OR 1.443; 95% CI: 1.050–1.983). However, age, rearing system, and climatic and environmental factors were not significantly associated with *T. gondii* seropositivity.

### 3.5. Seroprevalence of N. caninum in Goats

The overall seroprevalence was 15.5% (97 out of 627 samples) for IgG antibodies to *N. caninum* by ELISA. The true prevalence of *N. caninum* was 15.5%. The estimated *N. caninum* prevalence in goats in the temperate climate was 12.7% (45 out of 354 samples, Table 1). This study shows that the true prevalence was 12.7% for IgG antibodies against *N. caninum* in the temperate climate. The overall prevalence of *N. caninum* in the dry climate (Hidalgo) based on ELISA was 19.0% (52 out of 273, Table 2). The estimated *N. caninum* true prevalence in the dry climate performed on serum samples using ELISA was 19.0%.

### 3.6. Risk Factors of N. caninum in a Temperate Climate

The seroprevalence of *N. caninum* in different age groups ranged from 1.1% (1–2 years old) to 19.0% (≥2 years old; Table 1). The prevalence of the parasite in male goats, animals that had not undergone abortion, and those raised in the presence of dogs and in semi-intensive rearing systems was 12.4%, 13.4%, 16.6% and 13.0%, respectively, which was higher than those found in female goats (9.2%), animals with a history of abortion (11.9%), and animals raised on farms without dogs (7.0%) or in intensive rearing system (10.3%). Overall, the results of the bivariate logistic regression analysis indicate that there were no significant associations between the variables and *N. caninum*.

### 3.7. Risk Factors of N. caninum in a Dry Climate

Positive *N. caninum* IgG serum samples in goats were found in all three age groups, ranging from 2.9% to 33.8%; the highest seroprevalence was detected in animals that were ≤1 year old (Table 2). The highest seroprevalence of *N. caninum* was observed in female goats (23.1%), goats with no history of abortion (27.4%), goats raised in the presence of dogs (20.0%), and animals raised in intensive production systems (21.4%) in comparison with male animals (13.7%), goats with no history of abortion (12.8%), goats raised on farms without dogs (17.2%), and animals raised in intensive production systems (18.8%). Age was associated with *N. caninum* infection in animals: 1–2-year-old goats had a risk of being infected that was 2.209 times higher than that of goats ≤ 1 year old. The seroprevalence of *N. caninum* infection was not significantly influenced by sex, presence of dogs, rearing systems, or climate and environmental variables.

### 3.8. Risk Factors of N. caninum in Both Dry and Temperate Climates

The highest prevalence of *N. caninum* was observed in the ≤1-year-olds (22.0%), followed by the ≥2-year-olds (18.9%) and the 1–2-year-olds (1.9%). A summary of parasite seropositivity in two climate regions is presented in Table 4. The seroprevalence was higher in females (16.8%) than in males (13.1%). The highest percentage of *N. caninum* was detected in goats with no history of abortion (18.8%), in animals in contact with dogs (17.9%), and in animals raised in intensive systems (17.6%), compared with goats with a history of abortion (12.3%), in animals not in contact with dogs (11.4%), and in animals raised in semi-intensive systems (13.7%). The lowest estimated prevalence values were observed in the state of Puebla (12.73%, temperate climate) and the highest in Hidalgo (36.67%, dry climate).

The OR of Puebla state, which has a temperate climate (OR 1.616; 95% CI: 1.046–2.496), demonstrated that goats in the state were more vulnerable to Neosporosis than the animals in Hidalgo state, which has a dry climate. There was no significant association between *N. caninum* and age, sex, presence of dogs on the farms, rearing system, or climate and environment variables.

## 4. Discussion

*Seroprevalence of T. gondii in goats.* To our knowledge, the present study is the first to investigate *T. gondii* and *N. caninum* prevalence and epidemiological risk factors in two climate regions and two states of Mexico. Here, the overall prevalence of *T. gondii* based on ELISA in Mexican goats was 52.0%. The global seroprevalence of *T. gondii* in goats was estimated to be 27.49%, and the highest estimated seroprevalence was in Central America (62.15%), followed by Europe (31.53%), South America (29.76%), Africa (29.41%), and Asia (20.74%) [13]. A systematic review summarised that the global prevalence of *T. gondii* infection was 31.78%, indicating a wide distribution of this disease in the goat population. The *T. gondii* seroprevalence in goats was greater in Europe (38.88%) and Africa (37.89%) than on other continents [17]. However, comparing the data is challenging because of variations in diagnostic methods, farm and animal practices, and management factors, as well as the presence of domestic and wild felids [18].

*Risk factors of T. gondii in a temperate climate.* In this study, the prevalence of *T. gondii* in the temperate climate was 48.0%, similar to that reported in Spain (48.0%), although it is higher than the prevalence found in Italy (41.7%) [19], Lebanon (40%) [20], Taiwan (32.22%) [21], and China (16.76%) [22] and lower than another report in Italy (63.3%) [23], Algeria (53.26%) [24], and Switzerland (50.5%) [25].

Our study showed an association between female goats and risk of being *Toxoplasma* seropositive (OR 2.106; 95% CI: 1.322–3.355). A similar finding was reported in a temperate climate in China, which indicated a significant association between the seroprevalence of toxoplasmosis and female goats (OR 2.18; 95% CI: 1.40–3.39) [22]. In a systematic review, the seroprevalence in females was significantly higher than that in males. In general, females are more susceptible to infection by *T. gondii*, most likely due to their lower immunity during specific periods [5]. The higher female susceptibility to *T. gondii* may be associated with their increased lifespans for milk production and reproduction, while males are slaughtered at a younger age for meat supply [26].

*Risk factors of T. gondii in a dry climate.* The current survey shows a high prevalence of parasite infection (57.1%), although the antibody levels were higher than those reported in India (56.9%) [2], Egypt (38.28%) [27], Algeria (38.04%) [28], Pakistan (23.11–31.5%) [29,30], Mexico (12.6%) [31], and South Africa (10.5%) [32].

This study showed that a history of abortion is one of the most significant risk factors, consistent with previous reports in India [2] and Pakistan [29]. *Toxoplasma gondii* infections have been demonstrated to be a causative agent in cases of abortion, resulting in environmental contamination via cysts in placentas and foetuses. Inadequate disposal of these materials can infect cats, and thus increase subsequent oocyst shedding, which can persist in the environment for extended periods [2]. Bradyzoites and tissue cysts can survive for up to two weeks at 4 °C, but freezing kills them [33]. However, this finding was not consistent with the results of studies conducted in Algeria and Pakistan that reported that the prevalence of infection in goats was not significantly different according to the history of abortion [28,30]. In this study, age, sex, presence of cats, rearing system, and climatic and environmental factors were not significantly associated with *T. gondii* prevalence.

*Risk factors for T. gondii in both dry and temperate climates.* Based on our results, the variables of sex (OR 1.456), presence of cats (OR 1.970), and climate regions (OR 1.443) were significant risk factors for parasite exposure. Age, history of abortion, and rearing system were not significantly associated with *T. gondii* infection. Consistent with our results, Rodrigues et al. [13], who conducted a literature review of *T. gondii* in goats, found an association with female sex (OR 1.43) and the presence of cats (OR 2.22). Furthermore, Rodrigues et al. [13] found no significant differences between semi-intensive and intensive rearing and the presence/absence of other species.

Several authors have highlighted a significant association between the distribution of *Toxoplasma* infection and environmental and geoclimatic variables [8,10,34]. In our study, variables such as NDVI, LST, precipitation, and elevation, which are climatic and environmental parameters, were not statistically associated with *T. gondii* infection in dry and temperate climate regions. Human-induced climate change has an impact on heavy rainfall, thereby affecting the movement of oocysts of *T. gondii* [35]. *T. gondii* oocysts are remarkably resilient and can survive under a range of climate conditions and persist for long periods in faeces and soil after excretion by felids. The sporulated oocysts remain infective for 12–18 months in soil and water, and viable for longer periods of 24–54 months when stored at 4 °C in water. Environmental factors that result in the inactivity of oocysts include prolonged exposure to temperatures of −20 °C and high temperatures of up to 45 °C in water [1,36,37]. In the northwestern European region, the prolonged survival of oocysts is predominantly attributable to the presence of warmer and moister winters [9]. Furthermore, the consumption of water from domestic reservoirs is associated with the seroprevalence of *T. gondii* [38].

Bioclimatic classification schemes enable the extraction of relevant information from climate data, such as the Köppen climate classification, which is an integrated and convenient tool to identify the relationships between climate and the prevalence of parasite infection. The current survey shows a statistically significant association between both the dry and temperate climate regions (OR 1.443; 95% CI: 1.050–1.983), although a higher prevalence was observed in the dry climate than in the temperate ones. The occurrence, survival, distribution, and transmission of *T. gondii* are influenced by the characteristics of different climate regions [39].

*Seroprevalence of N. caninum in goats.* A total of 627 serum samples from goats in the Mexican states of Hidalgo and Puebla were used in the study, and the overall prevalence of *N. caninum* was 15.5%. A systematic review of 22,234 goats between 2004 and 2019 estimated the global seroprevalence of *N. caninum* to be 5.99%, with an estimated seroprevalence of 7.66% in the Americas. Differences in the estimated seroprevalence between countries and continents suggest that it varies among populations and may be associated with specific characteristics of each region, such as climatic factors and production systems of each population studied [6]. *T. gondii* oocysts exhibit greater resistance to freezing than to higher temperatures [39].

*Risk factors of N. caninum in a temperate climate*. The seroprevalence of *N. caninum* (12.7%) in goats in Puebla state in this study was higher than that reported in Spain (6.0%) [40], Italy 5.7% [41], China (3.9%) [42], and Switzerland (0.9%) [25].

This study, based on questionnaire responses and statistical analysis, revealed that age, sex, history of abortion, the presence of dogs, and rearing system variables were not key risk factors for *N. caninum* infection in goats in a temperate climate.

*Risk factors of N. caninum in a dry climate.* The mean prevalence of *N. caninum* in the dry climate (19.0%) was higher than that observed in India (10.99%) [2], Iran (10.8%) [43], and Mexico (3.3%) [31]. Here, the history of abortion was a significant risk factor for *N. caninum* infection. Similar findings regarding abortion were reported in Iran [43]. However, [2] reported no significant relationship between *N. caninum* prevalence and history of abortion in India. The increased rainfall in a dry environment has been demonstrated to compromise the success of gestation in *N. caninum*-infected cows [44].

*Risk factors for N. caninum in both dry and temperate climates.* In our study, a history of abortion was identified as a risk factor for parasite positivity (OR 1.645; 95% CI: 1.061–2.551). A systematic review reported similar results, indicating an association between *N. caninum* seropositivity and abortion (OR 3.07; 95% CI: 1.02–9.22) [6]. Here, there was no significant association between *N. caninum* and age, sex, presence of dogs, or rearing system. Rodrigues et al. [6] reported a similar finding that the age of goats was not associated with parasite infection; however, sex and the presence of dogs were considered risk factors for *N. caninum* seropositive goats.

In the present study, the transmission dynamics and persistence exhibited by dry and temperate climate regions were unaffected by variables such as NDVI, LST, precipitation, and elevation, which are characteristic climatic and environmental conditions. According to Rinaldi et al. [11], the risk of seropositivity decreases in proportion to increasing summer NDVI values determined for 3 km buffer zones around the farm. Mild temperatures and humidity favour the sporulation and survival of oocysts eliminated in dog faeces in the environment, which may increase the risk of exposure [33].

The specific environmental conditions of different climate regions can be used to predict the risk of *N. caninum* infection in ruminants [45,46]. The findings of this study show a significant association between the dry (Köppen classification type Bs) and temperate (Köppen classification type Cw) climate regions (OR 1.616; 95% CI: 1.046–2.496): a higher seroprevalence was found in the dry climate (Hidalgo state, 19.0%) than those in the temperate climate (Puebla state, 12.7%). A systematic review demonstrated a statistically significant relationship between geographic latitude and the prevalence of neosporosis in goats [6]. Two prior studies examining the impact of climate on the risk of *Neospora* in cattle in Germany and Italy identified the mean temperature in spring within a buffer zone surrounding the farm and the mean temperature in July in the municipality where the herd is situated as potential risk factors [11,47]. These observations can be explained by the effects of climate on the sporulation or survival of oocysts. For instance, a higher temperature may favour a faster sporulation of oocysts in fodder or in the environment [33].

## 5. Conclusions

The overall seroprevalences of *T. gondii* and *N. caninum* in the dry and temperate climate were 52.0% and 15.5%, respectively. *T. gondii* infection was significantly associated with sex, history of abortion, the presence of cats, and climate regions, while *N. caninum* prevalence in goats was significantly associated with age, history of abortion, and climate regions. The results of the present study demonstrate that *T. gondii* and *N. caninum* are highly prevalent in goats in different climate regions in two Mexican states. These results indicate that different risk factors are significantly associated with parasite prevalence in a dry and temperate climate. The obtained data may be useful for providing regionally adapted control measures and monitoring programmes to reduce abortion rates in goats and to avoid long-term economic losses.

## Figures and Tables

**Table 1:** The association of climatic, environmental, and risk factors with *Toxoplasma gondii* and *Neospora caninum* infection in a temperate climate region in Puebla state (*n* = 357).

Risk Factors		No. Tested	Positive No.	Prevalence (%)	Odds Ratio	95% CI	*p*-Value
*Toxoplasma gondii*	Age, year						
	≤1	87	20	23.0	1.000		
	1–2	88	20	22.7	0.113	0.062–0.205	<0.001
	≥2	179	130	72.6	0.111	0.061–0.201	<0.001
	Sex						
	Male	105	64	61.0	1.000		
	Female	249	106	42.6	2.106	1.322–3.355	0.002
	Abortion						
	No	186	41	22.0	1.000		
	Yes	168	129	76.8	0.085	0.052–0.141	<0.001
	Presence of Cats						
	No	143	53	37.1	1.000		
	Yes	211	117	55.5	0.473	0.306–0.731	0.001
	Rearing system						
	Intensive	39	17	43.6	1.000		
	Semi-Intensive	315	153	48.6	0.818	0.419–1.600	0.557
	Climate and environment						
	NDVI	354	170	48.0	1.000	1.000–1.000	0.867
	LST Day	354	170	48.0	1.000	0.999–1.002	0.710
	LST Noche	354	170	48.0	1.001	0.997–1.006	0.555
	Rainfall	354	170	48.0	0.230	0.000–188.964	0.668
	Elevation	354	170	48.0	1.000	0.999–1.001	0.391
*Neospora caninum*	Age, year						
	≤1	87	10	11.5	1.000		
	1–2	88	1	1.1	0.554	0.260–1.181	0.126
	≥2	179	34	19.0	0.049	0.007–0.364	0.003
	Sex						
	Male	105	13	12.4	1.000		
	Female	249	32	9.2	0.958	0.481–1.909	0.903
	Abortion						
	No	186	25	13.4	1.000		
	Yes	168	20	11.9	0.870	0.464–1.632	0.665
	Presence of Dogs						
	No	143	10	7.0	1.000		
	Yes	211	35	16.6	0.222	0.106–0.464	<0.001
	Rearing system						
	Intensive	39	4	10.3	1.000		
	Semi-Intensive	315	41	13.0	0.764	0.258–2.261	0.626
	Climate and environment						
	NDVI	354	45	12.7	1.000	1.000–1.000	0.867
	LST Day	354	45	12.7	1.000	0.999–1.002	0.710
	LST Noche	354	45	12.7	1.001	0.997–1.006	0.555
	Rainfall	354	45	12.7	0.230	0.000–188.964	0.668
	Elevation	354	45	12.7	1.000	0.999–1.001	0.391

**Table 2:** The association of climatic, environmental, and risk factors with *Toxoplasma gondii* and *Neospora caninum* infection in a dry climate region in Hidalgo state (*n* = 273).

Risk Factors		No. Tested	Positive No.	Prevalence (%)	Odds Ratio	95% CI	*p*-Value
*Toxoplasma gondii*	Age, year						
	≤1	77	11	14.3	1.000		
	1–2	68	41	60.3	0.038	0.018–0.084	<0.001
	≥2	128	104	81.3	0.350	0.181–0.677	0.002
	Sex						
	Male	117	70	59.8	1.000		
	Female	156	86	55.1	0.893	0.550–1.449	0.646
	Abortion						
	No	117	22	18.8	1.000		
	Yes	156	134	85.9	26.302	13.775–50.220	<0.001
	Presence of Cats						
	No	93	45	48.4	1.000		
	Yes	180	111	61.7	0.583	0.351–0.966	0.036
	Rearing system						
	Intensive	245	137	55.9	1.000		
	Semi-Intensive	28	19	67.9	0.601	0.261–1.381	0.230
	Climate and environment						
	NDVI	273	156	57.1	1.000	1.000–1.001	0.253
	LST Day	273	156	57.1	0.991	0.974–1.008	0.304
	LST Noche	273	156	57.1	0.998	0.974–1.022	0.849
	Rainfall	273	156	57.1	0.330	0.000–16.658	0.864
	Elevation	273	156	57.1	1.000	1.000–1.001	0.360
*Neospora* *caninum*	Age, year						
	≤1	77	26	33.8	1.000		
	1–2	68	2	2.9	2.209	1.155–4.224	0.017
	≥2	128	24	18.8	0.131	0.030–0.574	0.007
	Sex						
	Male	117	16	13.7	1.000		
	Female	156	36	23.1	0.528	0.277–1.007	0.053
	Abortion						
	No	117	32	27.4	1.000		
	Yes	156	20	12.8	2.560	1.376–4.763	0.003
	Presence of Dogs						
	No	93	16	17.2	1.000		
	Yes	180	36	20.0	0.528	0.277–1.007	0.053
	Rearing system						
	Intensive	245	46	18.8	1.000		
	Semi-Intensive	28	6	21.4	0.848	0.325–2.209	0.753
	Climate and environment						
	NDVI	273	52	19.0	1.000	1.000–1.001	0.703
	LST Day	273	52	19.0	0.993	0.971–1.015	0.518
	LST Noche	273	52	19.0	0.987	0.957–1.018	0.395
	Rainfall	273	52	19.0	0.21	0.000–12.465	0.715
	Elevation	273	52	19.0	1.000	1.000–1.001	0.428

**Table 3:** The association of climatic, environmental, and risk factors with *Toxoplasma gondii* seropositivity in a dry and temperate climate region in goats (*n* = 627).

Risk Factors	No. of Goats	Positive No.	Prevalence (%)	Odds Ratio	95% CI	*p*-Value
Age, year						
≤1	164	31	18.9	1.000		
1–2	156	61	41.0	0.073	0.045–0.113	<0.001
≥2	307	234	76.2	0.200	0.132–0.303	<0.001
Sex						
Male	222	129	58.1	1.000		
Female	405	197	48.6	1.465	1.053–2.038	0.024
Abortion						
No	303	63	20.8	1.000		
Yes	324	263	81.2	16.425	11.086–24.335	<0.001
Presence of Cats						
No	301	133	44.2	1.000		
Yes	326	193	59.2	1.970	1.419–2.734	<0.001
Rearing system						
Intensive	284	154	54.2	1.000		
Semi-Intensive	343	172	50.1	1.178	0.859–1.614	0.309
Climate and environment						
NDVI	627	326	52.0	1.000	1.000–1.000	0.280
LST Day	627	326	52.0	1.001	1.000–1.002	0.062
LST Noche	627	326	52.0	1.000	0.996–1.004	0.890
Rainfall	627	326	52.0	0.019	0.001–0.718	0.032
Elevation	627	326	52.0	0.999	0.998–1.00	0.078
State, Climate regions						
Hidalgo, Dry (Bs)	273	156	57.1			
Puebla, Temperate (Cw)	354	170	48.0	1.443	1.050–1.983	0.024

**Table 4:** The association of climatic, environmental, and risk factors with *Neospora caninum* seropositivity in a temperate and dry climate region in goats (*n* = 627).

Risk Factors	No. Tested	Positive No.	Prevalence (%)	Odds Ratio	95% CI	*p*-Value
Age, year						
≤1	164	36	22.0	1.000		
1–2	156	3	1.9	1.207	0.757–1.927	0.429
≥2	307	58	18.9	0.084	0.026–0.273	<0.001
Sex						
Male	222	29	13.1	1.000		
Female	405	68	16.8	0.745	0.466–1.191	0.218
Abortion						
No	303	57	18.8	1.000		
Yes	324	40	12.3	1.645	1.061–2.551	0.026
Presence of Dogs						
No	236	27	11.4	1.000		
Yes	391	70	17.9	0.340	0.210–0.546	<0.001
Rearing system						
Intensive	284	50	17.6	1.000		
Semi-Intensive	343	47	13.7	1.346	0.872–2.076	0.179
Climate and environment						
NDVI	627	97	15.5	1.000	1.000–1.001	0.288
LST Day	627	97	15.5	1.001	1.000–1.002	0.154
LST Noche	627	97	15.5	0.999	0.993–1.004	0.647
Rainfall	627	97	15.5	0.010	0.000–1.625	0.076
Elevation	627	97	15.5	0.999	0.998–1.001	0.273
State, Climate regions						
Hidalgo, Dry (Bs)	273	52	19.0			
Puebla, Temperate (Cw)	354	45	12.7	1.616	1.046–2.496	0.031

## Data Availability

Data are contained within the article.
